# Combination of an Antigen-Specific Therapy and an Immunomodulatory Treatment to Simultaneous Block Recurrent Autoimmunity and Alloreactivity in Non-Obese Diabetic Mice

**DOI:** 10.1371/journal.pone.0127631

**Published:** 2015-06-16

**Authors:** Georgia Fousteri, Tatiana Jofra, Roberta Di Fonte, Manuela Battaglia

**Affiliations:** Diabetes Research Institute (DRI), IRCCS San Raffaele Scientific Institute, Via Olgettina 58, Milan, Italy; University of Bremen, GERMANY

## Abstract

Restoration of endogenous insulin production by islet transplantation is considered a curative option for patients with type 1 diabetes. However, recurrent autoimmunity and alloreactivity cause graft rejection hindering successful transplantation. Here we tested whether transplant tolerance to allogeneic islets could be achieved in non-obese diabetic (NOD) mice by simultaneously tackling autoimmunity *via* antigen-specific immunization, and alloreactivity *via* granulocyte colony stimulating factor (G-CSF) and rapamycin (RAPA) treatment. Immunization with insB_9-23_ peptide alone or in combination with two islet peptides (IGRP_206-214 _and GAD_524-543_) in incomplete Freund’s adjuvant (IFA) were tested for promoting syngeneic pancreatic islet engraftment in spontaneously diabetic NOD mice. Treatment with G-CSF/RAPA alone or in combination with insB_9-23_/IFA was examined for promoting allogeneic islet engraftment in the same mouse model. InsB_9-23_/IFA immunization significantly prolonged syngeneic pancreatic islet survival in NOD mice by a mechanism that necessitated the presence of CD4^+^CD25^+^ T regulatory (Treg) cells, while combination of three islet epitopes was less efficacious in controlling recurrent autoimmunity. G-CSF/RAPA treatment was unable to reverse T1D or control recurrent autoimmunity but significantly prolonged islet allograft survival in NOD mice. Blockade of interleukin-10 (IL-10) during G-CSF/RAPA treatment resulted in allograft rejection suggesting that IL-10-producing cells were fundamental to achieve transplant tolerance. G-CSF/RAPA treatment combined with insB_9-23_/IFA did not further increase the survival of allogeneic islets. Thus, insB_9-23_/IFA immunization controls recurrent autoimmunity and G-CSF/RAPA treatment limits alloreactivity, however their combination does not further promote allogeneic pancreatic islet engraftment in NOD mice.

## Introduction

Type 1 diabetes (T1D) is an autoimmune disease characterized by the gradual loss of insulin-producing beta cells [1]. To date, several antigen-specific and antigen-non-specific immune interventions have been developed, which are assessed either as monotherapy or in combination in preventing and curing T1D [2, 3]. Results from preclinical models, such as the non-obese diabetic (NOD) mice, have highlighted the potential of antigen-specific approaches in controlling islet-specific autoimmunity efficiently and safely [4–6]. Clinical antigen-specific studies however, have so far failed to show the desired efficacy despite their good safety profile [7–9].

Reconstitution of the beta-cell compartment with pancreatic islet or whole pancreas transplantation is a promising strategy for the cure of T1D [10]. However, to protect from allograft rejection, generalized immunosuppression is needed. In the last decade, immunosuppression has been significantly refined and as a consequence, significant reduction in the incidence of acute rejection has been achieved [10, 11]. However, long-term graft survival has stagnated due to morbidity and mortality caused by the chronic use of immunosuppression [12].

Recurrent autoimmunity represents another formidable barrier to the success of pancreatic islet transplantation [13–16]. Studies in patients with T1D have shown that autoimmunity recurrence participates in graft loss, with very fast kinetic, reminiscent of a secondary, memory immune response [17–19]. It has also become apparent that recurrent autoimmunity is not effectively curtailed by the current immunosuppressive strategies that efficiently target alloreactivity [18–20]. On the basis of these findings, it has been proposed that the allogeneic and autoreactive immune responses against pancreatic islets are controlled by two independent mechanisms [21, 22].

We previously showed that insulin B chain 9–23 (insB_9–23_) immunization prevents T1D in NOD mice by increasing the number and invigorating the function of FOXP3^+^ T regulatory (Treg) cells [23, 24]. However, as with the majority of antigen-specific interventions in NOD mice, this treatment is ineffective after diabetes onset [23], suggesting that the remaining beta cell mass needs to be replenished to provide glycemic control. To date, few studies have addressed the effectiveness of antigen-specific immunization in controlling autoimmunity recurrence in NOD mice transplanted with syngeneic islets [25–27]. Given that the engrafted islets are subject to autoimmune destruction also in allogeneic transplantation, this approach might provide an additional advantage in prolonging graft survival. On the basis of this premise, we tested whether a single injection of insB_9–23_ can protect islet grafts placed under the kidney capsule of spontaneously diabetic NOD mice.

Quite remarkably, while various transplant tolerance-inducing protocols promote permanent allogeneic islet engraftment in autoimmune-resistant mouse strains, the same treatments have transient effect in NOD mice. For example, approaches that show good efficacy in inducing transplant tolerance in C57BL/6 recipients, i.e. treatments that induce significant reduction in alloreactive T cells (thymoglobulin, anti-CD3, anti-CD52) [28] and co-stimulation blockade monoclonal antibodies (mAbs) (anti-CTLA-4Ig, anti-CD80 and anti-CD86) [29, 30], have transient effect in NOD mice, suggesting that NOD mice are resistant to transplant tolerance induction [31–35].

We previously developed a tolerogenic treatment (granulocyte colony stimulating factor [G-CSF] and rapamycin [RAPA]) that induces robust allogeneic transplant tolerance in C57BL/6 mice *via* T regulatory type 1 (Tr1) cells [36], and was never tested in NOD mice. This protocol utilizes drugs that are routinely used in the clinic and are considered less invasive as compared to other immunosuppressive treatments. In this study we addressed whether G-CSF/RAPA can promote allogeneic islet engraftment in spontaneously diabetic NOD mice and investigated its efficacy in combination with antigen-specific immunization (insB_9–23_) to simultaneous tackle the recurrence of autoimmunity.

## Materials and Methods

### Mice

BALB/c and NOD/ShiLtJ (NOD) mice were purchased from Charles River (Calco, Italy). NOD mice were also bred in-house with breeders derived from Charles River and only mice from the F1 generation were used. All animals were housed under spf conditions. This study was approved by the Institutional Animal Care and Use Committee of the San Raffaele Scientific Institute (IACUC number: 511). All surgery was performed under Tribromoethanol (Avertin) anesthesia, and all efforts were made to minimize suffering.

### Islet transplant

Spontaneously diabetic NOD female mice with blood glucose levels ≥300mg/dl for two consecutive days were used as recipients for islet transplantation. Islets from male NOD (syngeneic) or female BALB/c (allogeneic) mice were isolated by collagenase digestion followed by density gradient separation, and approximately 600 pancreatic islets were transplanted under the kidney capsule as it was previously described for C57BL/6 recipients [36]. Since NOD mice did not develop T1D synchronously, two to three diabetic recipients were transplanted in each experiment and data were pooled. Transplantation was considered successful if non-fasting blood glucose levels returned to normal (<250 mg/dL) within 24 h after surgery. Blood glucose levels were monitored at least twice weekly with a monitoring system (OneTouch Ultra; LifeScan, Milpitas, CA, USA). Islet graft rejection was defined as recurrent hyperglycemia (≥250 mg/dL) for at least two consecutive days.

### Treatments

Transplanted mice were treated subcutaneously (s.c.) with 100 μg of insulin B chain 9–23 (insB_9–23_) peptide emulsified in incomplete Freund’s adjuvant (IFA) once as previously described [23]. InsB_9–23_, islet-specific glucose-6-phosphatase catalytic subunit related protein peptide 206–214 (IGRP_206–214_) and glutamic acid decarboxylase 65 peptide 524–543 (GAD_524–543_) (100μg each) were mixed together and emulsified in IFA prior to injection (combinatorial—combo- peptide treatment). G-CSF (Myelostim, rHuG-CSF, Italfarmaco) and RAPA (Rapamune; Wyeth Europe, Taplow, UK) were administered as previously described [36]. Briefly, G-CSF was diluted in PBS and injected subcutaneously (s.q.) at 200 mg/kg, whereas RAPA was diluted in water and administered by oral gavage at 1 mg/kg. G-CSF/RAPA treatment was given once a day for 30 days after transplantation. Anti-CD25 (clone PC61) monoclonal antibody (mAb) was given intravenously (i.v.) 500μg/dose per 5 daily doses [37]. Anti-IL-10 (clone JES5-2A5) mAb was given i.v. in three daily doses as follows: first dose 500 μg, second and third dose 250 μg [23].

### Flow cytometry staining

Surface cell staining was performed with anti-mouse CD3, CD4, CD25, CD8, CD62L, and CD44 mAbs (all from BD Pharmingen) following a 2.4G2 Fc block (anti-CD16/CD32 mAb) step. FOXP3 expression was tested with the FOXP3 staining kit (eBioscience, San Diego, CA) according to manufacturer’s instructions. Cells were acquired on a FACSCanto (BD Bioscience) and analyzed with FlowJo (Tree Star, USA) software.

### Immunohistochemistry

Kidneys were immersed in Tissue-Tek OCT (Bayer) and quick frozen on dry ice. Using cryomicrotome and Superfrost Plus slides (Fisher Scientific), 6-μm tissue sections were cut. Sections were fixed with 100% acetone at room temperature, and after washing in TBS, an avidin/biotin-blocking step was included (Vector Laboratories). Primary and secondary antibodies (Vector Laboratories) reacted with the sections for 60 min each, and colour reaction was obtained by sequential incubation with Vector Blue AP III and AEC (Vector Laboratories) as previously described [38]. Rat anti—mouse CD8a (Ly2)-biotin, rat anti—mouse CD4 (L3T4; BD Biosciences)-biotin and guinea pig anti-swine insulin (DAKO) were used. Goat anti-guinea pig AP was used to detect insulin [38].

### Statistics

Comparisons between groups were performed using the unpaired, two-tailed Student’s *t* test. Islet allograft survivals were determined with Kaplan-Meier survival curves and were compared with the log-rank test. In all cases the Prism software (GraphPad, USA) was used and, a two-tailed *P* value ≤ 0.05 was considered significant.

## Results

### InsB_9–23_/IFA peptide immunization prolongs syngeneic pancreatic islet graft survival in diabetic NOD mice

**Fig 1:**
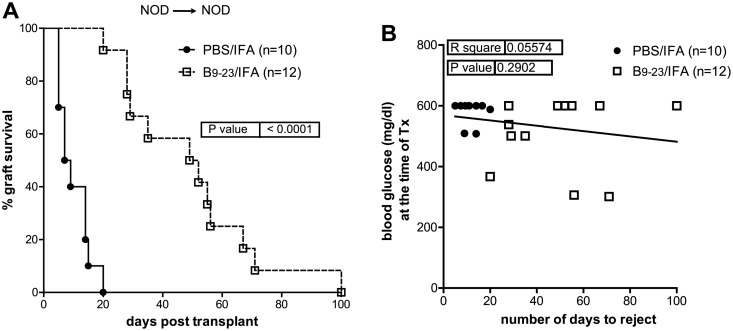
InsB_9–23_/IFA immunization temporarily controls recurrent autoimmunity in NOD mice. A, diabetic NOD mice were transplanted with islets from NOD donors (syngeneic) and treated with insB_9–23_/IFA or PBS/IFA. Percentage of islet graft survival after transplantation is shown. B, correlation between blood glucose levels at the time of transplant and the number of days of syngeneic islet engraftment for all mice. Each symbol represents one mouse. Survival curves were compared with the log-rank test. Correlation was done with Pearson coefficient. *P* values and R^2^ values are indicated in the graphs.

Syngeneic pancreatic islets were transplanted under the kidney capsule of spontaneously diabetic female NOD mice (NOD→NOD). A single immunization with insB_9–23_/IFA at the moment of transplant significantly prolonged islet graft survival, with a mean survival time (MST) of 50.5 (±25 SD) days, while control PBS/IFA-treated recipients remained normoglycemic for MST of 8 days (±10 SD) (Fig 1A). The mean blood glucose values of mice treated with insB_9–23_/IFA was 504.1 (±33.40 SD) mg/dl at the time of transplantation, while in the control was slightly higher, (580.5 mg/dl ±12.06 SD) (data not shown). However, islet graft survival did not correlate with blood glucose levels at the time of transplantation in any of the groups (Fig 1B), thus excluding any role for the blood glucose starting levels in the efficacy of the insB_9–23_/IFA immunization. These results show that antigen-specific immunization with insB_9–23_ transiently controls the recurrence of autoimmunity in NOD mice receiving syngeneic islets and its efficacy does not dependent on the blood glucose levels at the time of transplantation.

Histological examination of islets engrafted under the kidney capsule of the transplanted mice eleven days after transplantation showed reduced CD4^+^ and CD8^+^ T-cell infiltrates in the insB_9–23_/IFA-treated group as compared to control (S1 Fig and data not shown). Pancreas was also histologically examined in transplanted mice to determine any eventual endogenous islet function, and heavy insulitis with no insulin^+^ islets were identified (data not shown). On the contrary, grafts from insB_9–23_/IFA-treated mice analyzed at the time of rejection showed insulin^+^ cells surrounded by T-cell infiltrates (S1 Fig). These results show that insB_9–23_/IFA treatment promotes syngeneic pancreatic islet transplantation.

### Depletion of CD25^+^ T cells in insB_9–23_/IFA-immunized mice prevents syngeneic islet graft tolerance and results in rejection

We previously showed that insB_9–23_/IFA immunization mice increases the frequency and number of FOXP3^+^ Treg cells in prediabetic NOD mice approximately two weeks after treatment and that CD4^+^CD25^+^ T cells from protected mice can transfer tolerance into new prediabetic NOD recipients [23]. To address whether insB_9–23_/IFA treatment increases FOXP3^+^ Treg cells also in the syngeneic islet transplant setting, peripheral blood, kidney draining lymph nodes (dLN), graft and spleen cells from insB_9–23_/IFA-treated and control mice were analyzed eleven days after transplantation. The frequency and number of FOXP3^+^ Treg cells within CD4^+^ T cells was similar between insB_9–23_/IFA—immunized and control recipients in the blood, spleen and graft, whereas they were slightly increased in the kidney dLN of insB_9–23_/IFA—immunized recipients (S2 Fig). Thus, insB_9–23_/IFA treatment did not significantly increase FOXP3^+^ Treg cells frequency in syngeneically transplanted NOD mice eleven days after transplantation.

**Fig 2:**
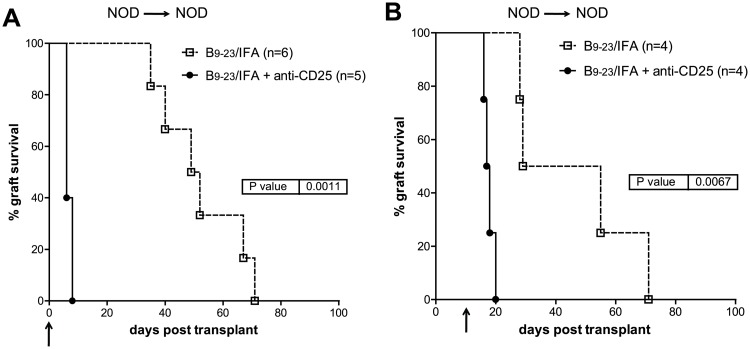
InsB_9–23_/IFA treatment efficacy is dependent on CD4^+^CD25^+^ Treg cells. Spontaneously diabetic female NOD mice were transplanted with syngeneic islets and with insB_9–23_/IFA. A, some mice were received anti-CD25 mAb administration injected at the same time of transplantation or B, 10 days after transplantation. Arrows indicate the time when anti-CD25 mAb treatments initiated. Overall graft survival is shown. Survival curves were compared with the log-rank test. *P* values are indicated in the graphs.

The fact that the frequency and number of FOXP3^+^ Treg cells did not increase after insB_9–23_/IFA immunization does not exclude that CD4^+^CD25^+^ Treg cells mediated syngeneic graft tolerance in insB_9–23_/IFA-immunized mice. To this end, anti-CD25 depleting mAb [39] was administered to insB_9–23_/IFA-treated NOD mice either at the time of transplantation or ten days later. In both cases, immediate and quite synchronous graft rejection was observed (Fig 2). This data shows that CD4^+^CD25^+^ T cells are essential in recipient mice both at the time of transplantation and ten days later to promote syngeneic islet engraftment after insB_9–23_/IFA immunization.

### Combination of three islet epitopes, insB_9–23_, GAD65_524–543_ and IGRP_206–214_ is less efficient than insB_9–23_ alone at promoting syngeneic pancreatic islet engraftment in NOD mice

**Fig 3:**
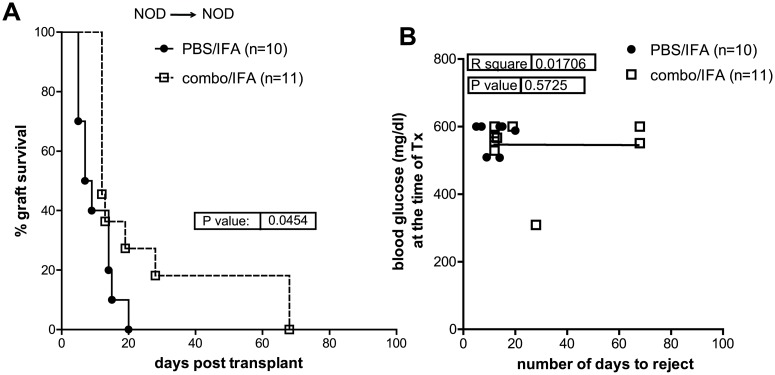
Combination therapy with InsB_9–23_, IGRP_206–214_ and GAD_524–543_ is less efficacious in promoting syngeneic islet transplant tolerance in NOD mice. A, diabetic NOD mice were transplanted with syngeneic islets. Recipients were treated with a mix of 3 islet peptides, insB_9–23_, IGRP_206–214_ and GAD_524–543_/IFA and monitored for graft engraftment. Graph shows the percentage of islet graft survival after transplantation. Control, PBS/IFA-treated, mice were pooled from different experiments and used as reference in all experiments (see Materials and Methods). B, correlation between blood glucose levels at the time of transplant and the number of days of syngeneic islet engraftment for all mice. Survival curves were compared with the log-rank test. Correlation was done with Pearson coefficient. *P* and R^2^ values are indicated in the graphs.

It has been proposed that combination of multiple islet epitopes might improve the outcome of antigen-specific tolerance by targeting more effector T cells and increasing the spectrum of Treg cell antigen specificity [9]. Here we addressed whether combination of insB_9–23_, GAD65_524–543_ and IGRP_206–214_ in IFA (combo/IFA) is more able than insB_9–23_/IFA alone to promote syngeneic pancreatic islet survival in NOD mice. Combo/IFA treatment did not provide additional advantage in controlling syngeneic islet graft rejection as compared to insB_9–23_/IFA mono-peptide immunization (Fig 3A). MST in combo/IFA-treated mice was of 12 (±30 SD) days (Fig 3A), much lower as compared to MST insB_9–23_/IFA-treated mice (MST: 50 [±25 days SD]) as shown in Fig 1A. All recipient mice had ≥300mg/dl at the time of transplantation and no correlation between blood glucose values and rejection time was seen (Fig 3B). Thus, treatment with combo/IFA is less efficacious as compared to insB_9–23_ alone in controlling rejection of syngeneic islet grafts in NOD mice.

### G-CSF/RAPA treatment prolongs allogeneic pancreatic islet survival in NOD mice in an IL-10-dependent manner

**Fig 4:**
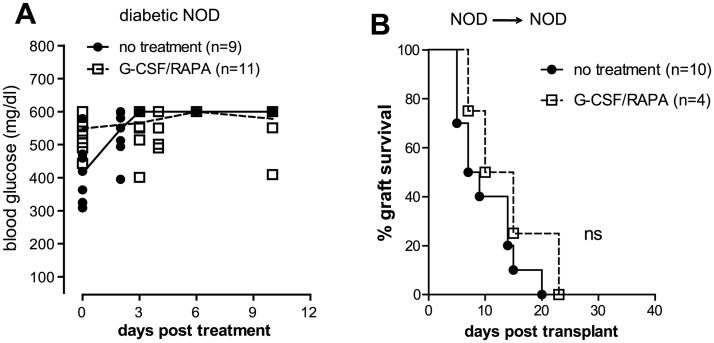
G-CSF/RAPA treatment does not reverse diabetes in NOD mice and does not control the recurrence of autoimmunity. A, diabetic NOD mice were treated with G-CSF/RAPA and monitored for diabetes progression. Graph shows the blood glucose values of mice prior and after treatment over time. B, diabetic NOD mice were transplanted with islets from NOD donors and treated with G-CSF/RAPA. Graph shows the percentage of islet graft survival after transplantation. Differences between treated and untreated mice are not statistically significant (ns).

We previously showed that a 30-day regimen composed of G-CSF and RAPA induces robust allogeneic transplant tolerance in chemically-induced diabetic C57BL/6 mice transplanted with BALB/c islets (BALB/c→C57BL/6) *via* Tr1 cells [36]. Here we addressed the tolerogenic effect of the same treatment in spontaneously diabetic NOD mice receiving islets from BALB/c donors. Initially, we tested whether G-CSF/RAPA treatment has any effect on anti-islet autoreactivity in the absence of islet replacement. G-CSF/RAPA did not reverse disease in overtly diabetic NOD mice (Fig 4A). In addition, G-CSF/RAPA treatment did not control syngeneic islet rejection (NOD→NOD) (Fig 4B). Thus, G-CSF/RAPA treatment is unable to control islet-specific autoreactivity since both un-transplanted and syngeneically transplanted NOD mice treated with this regimen were not cured from T1D.

**Fig 5:**
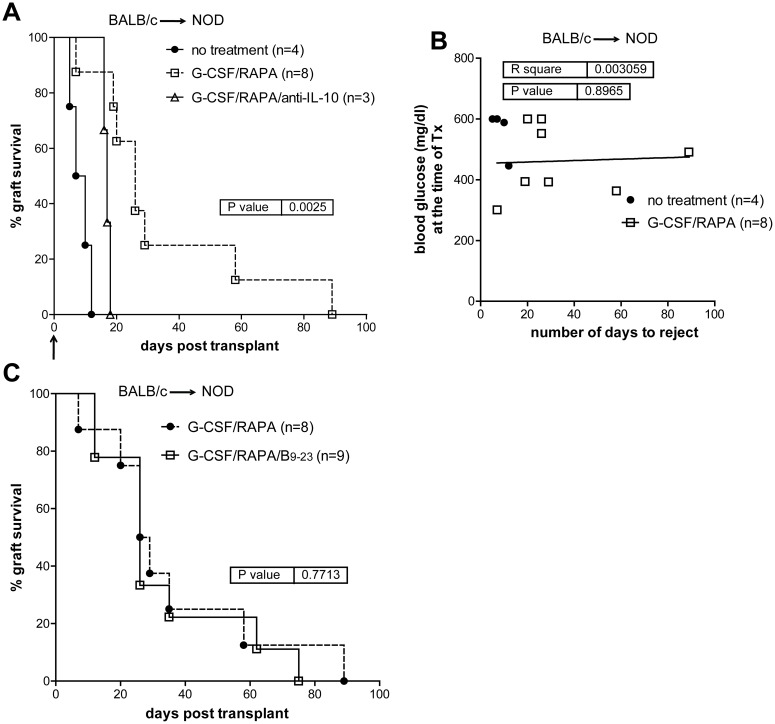
G-CSF/RAPA treatment induces transplant tolerance to allogeneic islets in NOD mice that depends on IL-10 production. A, spontaneously diabetic female NOD mice were transplanted with allogeneic islets from BALB/c donors. Recipients were treated with G-CSF/RAPA and monitored for graft survival. Anti-IL-10 was administered in NOD mice transplanted and treated with G-CSF/RAPA. Arrow indicates the time when anti-IL-10 mAb treatments initiated. Overall graft survival is shown. Graph shows the percentage of islet graft survival after transplantation. B, correlation between blood glucose levels at the time of transplant and the number of days of syngeneic islet engraftment. C, graph shows the percentage of islet graft survival in G-CSF/RAPA vs. G-CSF/RAPA/insB_9–23_/IFA-treated recipients. Survival curves were compared with the log-rank test. *P* values are indicated in the graphs.

Quite unexpectedly, implementing G-CSF/RAPA in NOD mice receiving allogeneic islets from BALB/c donors (BALB/c→NOD) led to transient but significantly prolonged islet allograft survival (MST: 26 ±30 days) as compared to untreated recipients (MST: 8.5 ±2 SD days) (Fig 5A). The effect of G-CSF/RAPA-treatment in NOD mice transplanted with allogeneic islets was dependent on IL-10 production, since allogeneic pancreatic islets were rejected if mice were treated with anti-IL-10 mAb at the time of transplantation (Fig 5A). Also here, no correlation between blood glucose levels and MST was observed (Fig 5B).

Thus far our data indicates that insB_9–23_/IFA immunization controls autoimmunity both in prediabetic [23] and diabetic NOD mice transplanted with syngeneic islets. Moreover, G-CSF/RAPA treatment temporarily controls allograft rejection in diabetic NOD mice. Next, we sought to combine the two approaches to determine whether they could impart a synergistic effect leading to indefinite allogeneic islet graft survival and cure T1D in spontaneously diabetic NOD mice. This combination did not provide any significant improvement in graft survival as compared to G-CSF/RAPA treatment alone (Fig 5C). Together, these results show that the combination of two approaches that act on different “arms” of immune regulation, i.e. autoimmunity and alloreactivity, does not further prolong pancreatic islet allograft survival in spontaneously diabetic NOD mice.

## Discussion

Pancreatic islet transplantation is considered a promising approach for the cure of T1D. To efficiently promote allogeneic islet transplant tolerance in patients with T1D two fronts need to be simultaneously tackled: alloreactivity and the recurrent autoimmunity. In this study we show that insB_9–23_/IFA immunization, a treatment that efficiently prevents but does not revert diabetes in NOD mice [23], is able to transiently control the recurrence of autoimmunity. Furthermore, G-CSF/RAPA, a safe and clinically relevant protocol that induces robust allogeneic islet transplant tolerance in C57Bl/6 mice [36], significantly prolongs allogeneic islet graft survival also in diabetic NOD mice. However, combination of the two approaches does not have a synergistic effect as it does not succeed inducing permanent islet allograft survival in NOD mice or prolonging the effect of G-CSF/RAPA treatment.

We previously showed that a single injection with insB_9–23_/IFA prevents the development of T1D in NOD mice by augmenting the frequency and number of CD4^+^CD25^+^FOXP3^+^ Treg cells [23]. In this study, insB_9–23_/IFA immunization controlled the recurrence of autoimmunity also *via* FOXP3^+^ Treg cells, although it did not significantly increase their number. Today, stem cell therapy, cellular reprogramming and whole-organ bioengineering are in the pipeline, opening new horizons toward an efficient, immunosuppression-free syngeneic beta cell replacement (reviewed in [40]). However, the control of recurrent autoimmunity remains a great challenge. Our new findings show that insB_9–23_/IFA peptide immunization is a promising approach that could be implemented to control recurrent autoimmunity improving the survival of syngeneic islet grafts.

In the present syngeneic islet transplantation model, insB_9–23_/IFA peptide immunization was effective in prolonging syngeneic islet graft survival in NOD mice, but did not provide permanent protection from diabetes recurrence. Approaches such as antigen re-injection and combination of multiple antigens are considered possible ways to improve the efficacy of antigen-specific therapies. Although future studies will address whether antigen re-injection can prolong the efficacy, we previously showed that factors such as too frequent immunization could negatively impact the efficacy of antigen-specific therapy [24]. To improve the tolerogenic outcome of insB_9–23_/IFA immunization, we sought to combine it with two other islet epitopes (GAD65_524–543_ and IGRP_206–204_—combo/IFA), known to be targeted in NOD mice and to prevent diabetes once administered *via* tolerogenic routes [41–45]. Rather unexpectedly, immunization with combo/IFA was less efficient as compared to insB_9–23_/IFA alone in controlling recurrent autoimmunity.

Although multiple islet-epitope immunization has not been extensively tested in NOD mice, data from mouse models of multiple sclerosis has shown that combination of multiple myelin epitopes leads to increased efficacy [46]. To our knowledge combo islet-specific peptide immunizations have not been extensively tested in NOD mice, with an exception of one study in new-born mice, which unexpectedly induced precocious islet-specific autoreactivity [47]. Therefore, more combinatorial antigen-specific studies need to be done at a preclinical level, since a number of factors, i.e. the nature of the antigen, the dose, the number of doses and frequency, the route and/or mode of administration seem to influence the efficacy and safety of these experimental treatments.

In the syngeneic islet transplant setting used here, NOD male mice served exclusively as donors and spontaneously diabetic female mice as recipients (male-to-female NOD). Male mice are known to have reduced number of infiltrating autoreactive T cells in the pancreas. As a consequence, the number of diabetogenic leukocytes that could pass from the donor (passenger) and negatively influence the treatment’s efficacy, would be less when compared to a female-to female NOD setting. To address this, more mechanistic studies on the nature and antigen-specificity of the graft-rejecting T cells are necessary. These studies will define whether passenger diabetogenic insB_9–23_-specific T cells are the cause of graft rejection in insB_9–23_/IFA-treated mice, and will determine what other autoreactive T cell specificities participate in graft rejection. This knowledge is pivotal as it will guide us to more accurately intervene and block the recurrence of autoimmunity.

We previously showed that G-CSF/RAPA treatment induces robust transplant tolerance in C57BL/6 mice *via* Tr1 cells [36]. In this study, the same treatment promoted allogeneic islet engraftment also in NOD mice, but was less efficient as compared to C57BL/6 recipients, confirming the resistance of NOD mice to transplant tolerance induction [33–35]. It is conceivable that autoimmunity played an important role in graft loss in NOD recipients, since G-CSF/RAPA treatment was inefficient to reverse diabetes or to prolong syngeneic islet graft survival in NOD mice, suggesting that it could not abrogate autoimmunity. These results support previous observations, sustaining that autoreactivity and alloreactivity are different processes, controlled by distinct and possibly non-overlapping mechanisms [22]. The reason behind this division is not yet clear. Most probably the expression of major histocompatibility complex (MHC) on beta cells or other passenger cells, including antigen presenting cells, plays significant role in determining how islet grafts are destroyed, i.e. via autoreactive or alloreactive T cells. As a consequence, certain therapies seem have specific effect by blocking the activation of T cells that are specific for self or allo antigens [20, 35].

Transplantation of allogeneic islets into spontaneously diabetic NOD mice represents the situation most frequently encountered in the clinic today. Usually MHC miss-matched islets are transplanted in diabetic patients, which are exposed to graft rejection due to alloreactivity and recurrence of autoimmunity. Thus, to simultaneous treat both alloreactivity and autoimmunity, NOD recipients of BALB/c islets were treated with G-CSF/RAPA and insB_9–23_/IFA. This combination treatment did not further prolong pancreatic islet allograft survival, showing that the two treatments did not have an additive effect. Possibly, deep analysis of the T cell specificity might provide further insights into the mechanisms leading to graft rejection and lack of synergy in the treated mice.

In summary, here we tested the efficacy of antigen-specific immunotherapy and G-CSF/RAPA treatment in promoting pancreatic islet survival in diabetic NOD mice. Both approaches show significant but transient efficacy in controlling recurrent autoimmunity and alloreactivity and additional functional studies are necessary to understand how we can improve transplant tolerance in NOD mice and how the efficient preclinical protocols can be translated to humans.

## Conclusions

In this study, we utilized a stringent model of allograft islet transplantation into spontaneously diabetic NOD mice to test a combination immunotherapy that targets both allogeneic and autoimmune responses. Antigen-specific therapy, which we previously showed to delay diabetes development in pre-diabetic NOD mice (insB_9–23_/IFA) [23], was combined with an immunomodulatory treatment (G-CSF/RAPA) that we developed for controlling allograft rejection [36]. Our findings show that insulin antigen-specific therapy delays autoimmune-mediated graft rejection and G-CSF/RAPA treatment delays islet allograft, but the combination of the two approaches does not enhance graft survival in the BALB/c→NOD allogeneic model of islet transplantation. We also provide some insight into the mechanisms behind the two therapies. Our careful and stringent assessment of combinatorial immunotherapies uncovers a critical problem in the field of islet transplantation—antigen-specific therapies that work in early stages of disease development might not be optimal for preventing anti-islet memory autoimmune responses. The findings in our study underscore our current limited understanding of the immune responses involved in islet transplant rejection and support further studies of the mechanisms, antigens being targeted, and cell types involved in islet rejection.

## Supporting Information

S1 Fig InsB_9–23_/IFA treatment decreases autoimmune lymphocytic infiltration in transplanted islet grafts. A, diabetic NOD mice were transplanted with islets from NOD donors and treated s.c. once with insB_9–23_/IFA or PBS/IFA. Eleven days post transplantation mice were killed and islets transplanted under the kidney capsule were histologically examined for the presence of CD4^+^ T cells. Images show the representative staining for insulin (blue) / CD4 (red) in one control mouse (treated with PBS/IFA) and one mouse treated with insB_9–23_/IFA (magnification 20x) (three mice per group). B, once turned diabetic, insB_9–23_/IFA-treated mice were killed and islet graft infiltration was assessed for the presence of CD4^+^ and CD8^+^ T cells. Histology from one representative mouse that rejected the islet graft 56 days post-transplant is shown.
(TIF)

S2 Fig InsB_9–23_/IFA treatment does not alter the peripheral FOXP3^+^ Treg cell frequency and number. Transplanted NOD mice were treated once s.c. with insB_9–23_/IFA or PBS/IFA (control). Flow cytometry was used to determine the frequency and total number of CD4^+^FOXP3^+^ Treg cells in the blood, kidney draining lymph nodes (KdLN), graft and spleen 11 days after transplantation. A, Representative flow cytometry plots depict the frequency of FOXP3^+^ CD4^+^ T cells in blood and spleen of PBS/IFA and insB_9–23_/IFA-treated mice. 8-12-wk-old NOD unmanipulated normoglycemic mice were used as controls. B-F, the percentage of FOXP3^+^ (Treg) cells gated on CD4^+^ T cells in blood (B), KdLN (C) and graft (D), as well as the percentage (E) and total number (F) of Treg cells in the spleen was assessed 11 days after transplantation. Unmanipulated, non-diabetic 8–12 wk-old NOD mice were used as additional control.
(TIF)
