# Supplementary figures and images for: Combination of an Antigen-Specific Therapy and an Immunomodulatory Treatment to Simultaneous Block Recurrent Autoimmunity and Alloreactivity in Non-Obese Diabetic Mice
Source: PLoS One. 2015 Jun 16;10(6):e0127631. doi: 10.1371/journal.pone.0127631 (PMC4469694; doi:10.1371/journal.pone.0127631)

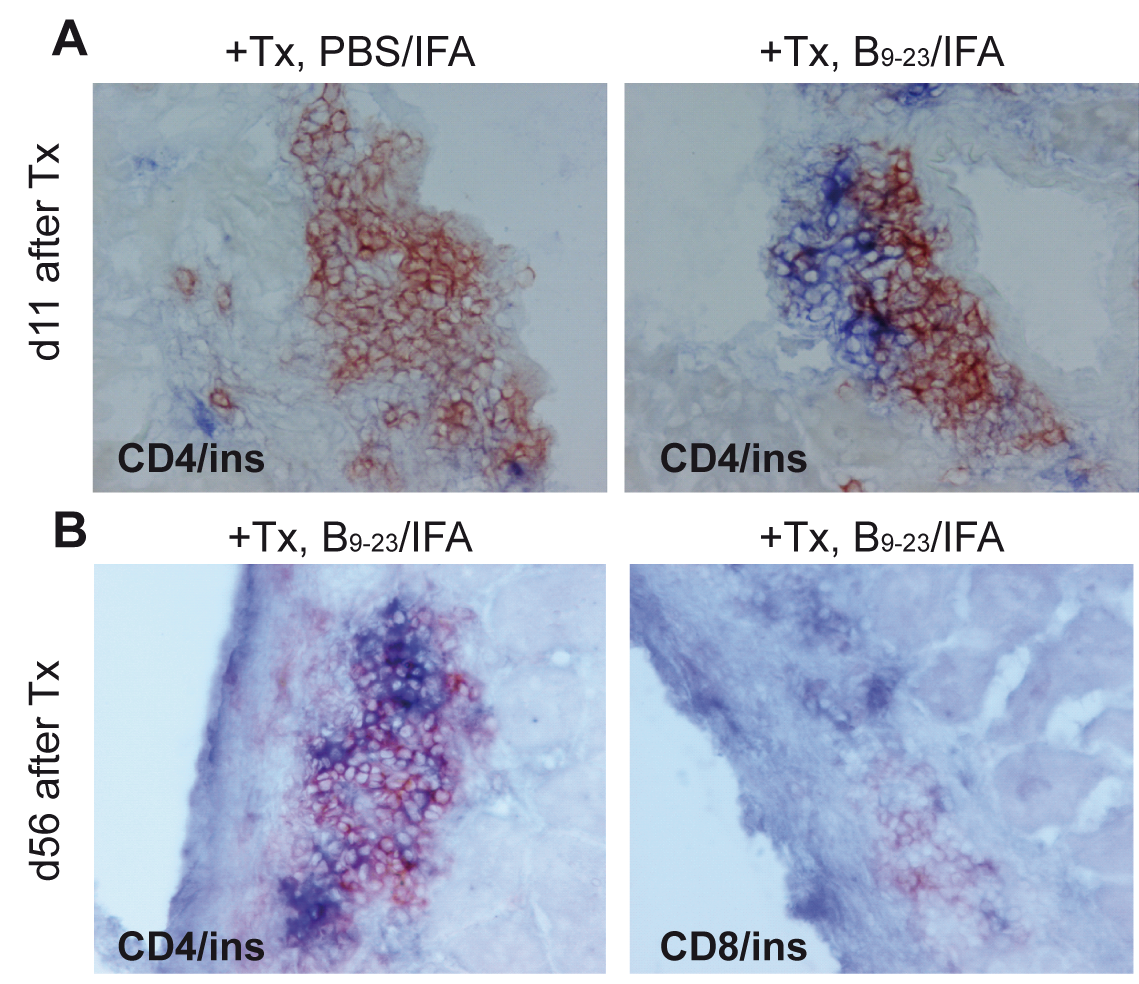

Supplement: S1 Fig — A, diabetic NOD mice were transplanted with islets from NOD donors and treated s.c. once with insB9–23/IFA or PBS/IFA. Eleven days post transplantation mice were killed and islets transplanted under the kidney capsule were histologically examined for the presence of CD4+ T cells. Images show the representative staining for insulin (blue) / CD4 (red) in one control mouse (treated with PBS/IFA) and one mouse treated with insB9–23/IFA (magnification 20x) (three mice per group). B, once turned diabetic, insB9–23/IFA-treated mice were killed and islet graft infiltration was assessed for the presence of CD4+ and CD8+ T cells. Histology from one representative mouse that rejected the islet graft 56 days post-transplant is shown. (TIF) [file pone.0127631.s001.tif]

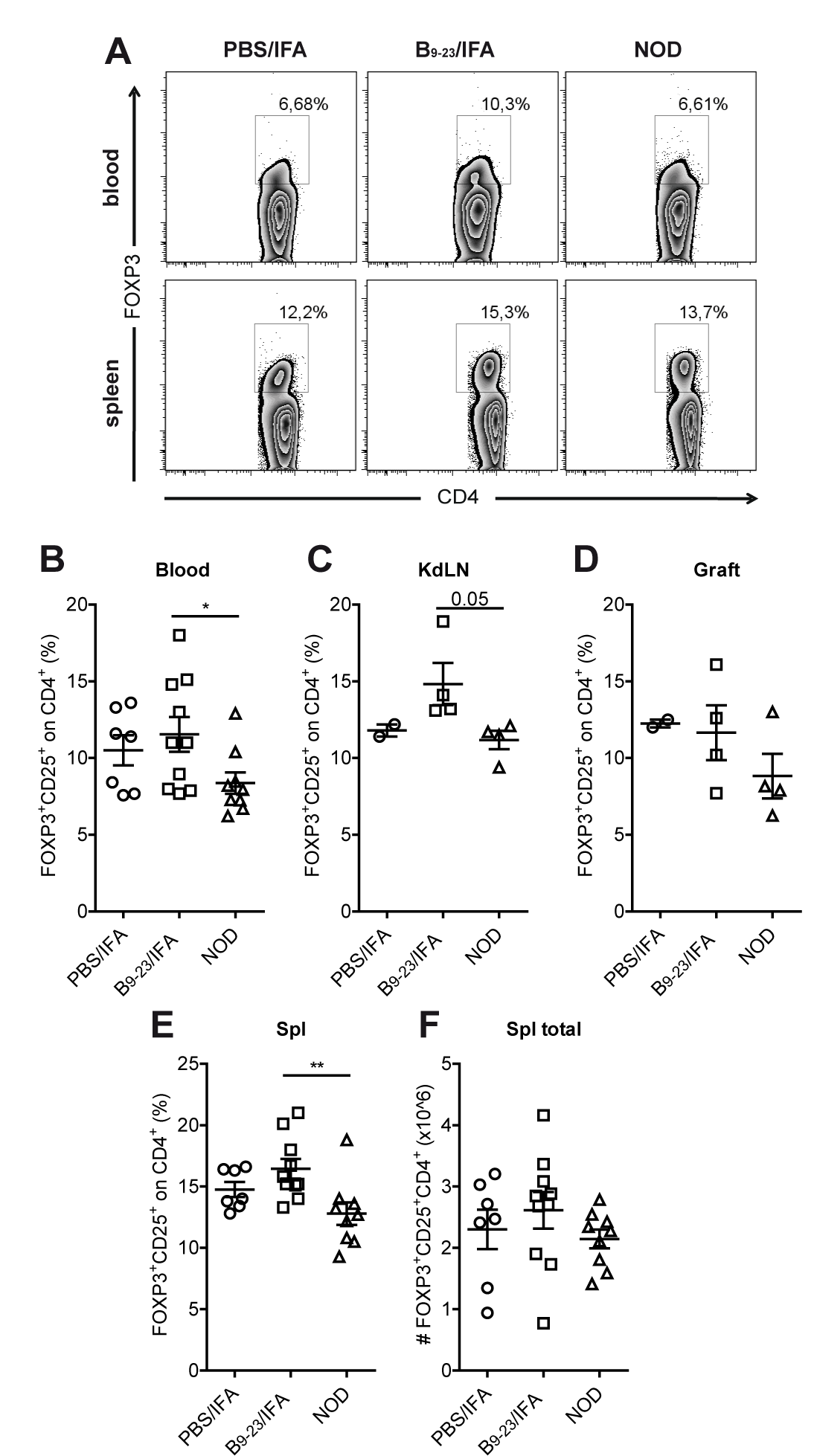

Supplement: S2 Fig — Transplanted NOD mice were treated once s.c. with insB9–23/IFA or PBS/IFA (control). Flow cytometry was used to determine the frequency and total number of CD4+FOXP3+ Treg cells in the blood, kidney draining lymph nodes (KdLN), graft and spleen 11 days after transplantation. A, Representative flow cytometry plots depict the frequency of FOXP3+ CD4+ T cells in blood and spleen of PBS/IFA and insB9–23/IFA-treated mice. 8-12-wk-old NOD unmanipulated normoglycemic mice were used as controls. B-F, the percentage of FOXP3+ (Treg) cells gated on CD4+ T cells in blood (B), KdLN (C) and graft (D), as well as the percentage (E) and total number (F) of Treg cells in the spleen was assessed 11 days after transplantation. Unmanipulated, non-diabetic 8–12 wk-old NOD mice were used as additional control. (TIF) [file pone.0127631.s002.tif]
